# Genotoxic effects in occupational exposure to formaldehyde: A study in anatomy and pathology laboratories and formaldehyde-resins production

**DOI:** 10.1186/1745-6673-5-25

**Published:** 2010-08-20

**Authors:** Susana Viegas, Carla Nunes, Joana Malta-Vacas, Mário Gomes, Miguel Brito, Paula Mendonça, João Prista

**Affiliations:** 1 Environmental Health Department. Escola Superior de Tecnologia da Saúde de Lisboa - Instituto Politécnico de Lisboa. Lisbon, Portugal; 2 Anatomy and Pathology Department. Escola Superior de Tecnologia da Saúde de Lisboa - Instituto Politécnico de Lisboa. Lisbon, Portugal; 3 CIESP - Centro de Investigação e Estudos em Saúde Pública (ENSP/UNL) ENSP - Escola Nacional de Saúde Pública - Universidade Nova de Lisboa. Lisbon, Portugal; 4 Biology Department. Escola Superior de Tecnologia da Saúde de Lisboa - Instituto Politécnico de Lisboa. Lisbon, Portugal; 5 Chemistry Department. Escola Superior de Tecnologia da Saúde de Lisboa - Instituto Politécnico de Lisboa. Lisbon. Portugal and REQUIMTE/CQFB, Faculty of Sciences and Technology, Universidade Nova de Lisboa and SINTOR-UNINOVA, Monte de Caparica, Portugal; 6 Environmental and Occupational Health Department and CIESP - Centro de Investigação e Estudos em Saúde Pública (ENSP/UNL) ENSP - Escola Nacional de Saúde Pública - Universidade Nova de Lisboa. Lisbon, Portugal

## Abstract

**Background:**

According to the Report on Carcinogens, formaldehyde ranks 25^th ^in the overall U.S. chemical production, with more than 5 million tons produced each year. Given its economic importance and widespread use, many people are exposed to formaldehyde environmentally and/or occupationally. Presently, the International Agency for Research on Cancer classifies formaldehyde as carcinogenic to humans (Group 1), based on sufficient evidence in humans and in experimental animals. Manyfold *in vitro *studies clearly indicated that formaldehyde can induce genotoxic effects in proliferating cultured mammalian cells. Furthermore, some *in vivo *studies have found changes in epithelial cells and in peripheral blood lymphocytes related to formaldehyde exposure.

**Methods:**

A study was carried out in Portugal, using 80 workers occupationally exposed to formaldehyde vapours: 30 workers from formaldehyde and formaldehyde-based resins production factory and 50 from 10 pathology and anatomy laboratories. A control group of 85 non-exposed subjects was considered. Exposure assessment was performed by applying simultaneously two techniques of air monitoring: NIOSH Method 2541 and Photo Ionization Detection equipment with simultaneously video recording. Evaluation of genotoxic effects was performed by application of micronucleus test in exfoliated epithelial cells from buccal mucosa and peripheral blood lymphocytes.

**Results:**

Time-weighted average concentrations not exceeded the reference value (0.75 ppm) in the two occupational settings studied. Ceiling concentrations, on the other hand, were higher than reference value (0.3 ppm) in both. The frequency of micronucleus in peripheral blood lymphocytes and in epithelial cells was significantly higher in both exposed groups than in the control group (p < 0.001). Moreover, the frequency of micronucleus in peripheral blood lymphocytes was significantly higher in the laboratories group than in the factory workers (*p *< 0.05). A moderate positive correlation was found between duration of occupational exposure to formaldehyde (years of exposure) and micronucleus frequency in peripheral blood lymphocytes (r = 0.401; *p *< 0.001) and in epithelial cells (r = 0.209; *p *< 0.01).

**Conclusions:**

The population studied is exposed to high peak concentrations of formaldehyde with a long-term exposure. These two aspects, cumulatively, can be the cause of the observed genotoxic endpoint effects. The association of these cytogenetic effects with formaldehyde exposure gives important information to risk assessment process and may also be used to assess health risks for exposed workers.

## Background

Formaldehyde (CH_2_O), the most simple and reactive of all aldehydes, is a colorless, reactive and readily polymerizing gas at room temperature [1]. It has a pungent suffocating odor that is recognized by most human subjects at concentrations below 1 ppm [2].

According to the Report on Carcinogens, formaldehyde (FA) ranks 25^th ^in the overall U.S. chemical production, with more than 5 million tons produced each year [3]. FA annual production rises up to 21 million tons worldwide and it has increased in China, for example, in the recent years, with 7,5 million tons produced in 2007. Given its economic importance and widespread use, many people are exposed to FA environmentally and/or occupationally [4]. According to the International Information System on Occupational Exposure to Carcinogens (CAREX), in the period between 1990 and 1993, 36,000 workers were occupationally exposed to FA in Portugal [5].

Occupational exposure involves not only workers in direct production of FA and products containing it, but also in industries utilizing these products, such as those related with construction and household [1]. The most extensive use of FA is in production of resins with urea, phenol and melamine, and also polyacetal resins. These products are used as adhesives in manufacture of particle-board, plywood, furniture and other wood products [2]. FA is also used in cosmetics composition and has an important application as a disinfectant and preservative, reason why relevant workplace exposure may also occur in pathology and anatomy laboratories and in mortuaries [1,2,6].

Human studies have shown that chronic exposure to FA by inhalation is associated with eye, nose and throat irritation [7]. Mostly important, several studies report a carcinogenic effect in humans after chronic exposure to FA, in particular an increased risk for nasopharyngeal cancer [8-12]. Since 2006, International Agency for Research on Cancer (IARC) classifies FA as carcinogenic to humans (Group 1), based on sufficient evidence in humans and in experimental animals [2]. IARC also concluded that there is a "strong but not sufficient evidence for a causal association between leukemia and occupational exposure to formaldehyde".

With actual scientific evidence we can conclude that, regarding to risk estimation, local toxic effects at site of first contact seem to be the most relevant health effects [2,7,13].

Manifold *in vitro *studies clearly indicated that FA can induce genotoxic effects in proliferating cultured mammalian cells [2]. Furthermore, some *in vivo *studies detected changes in epithelial cells (oral and nasal) and in peripheral lymphocytes related to FA exposure [13,14].

Frequency of micronucleus (MN) in buccal and/or nasal mucosa cells is being used to investigate local genotoxicity. According to reports concerning experimental genotoxicity studies, MN are the most sensitive genetic endpoints for detection of FA induced genotoxicity [15]. Thus, MN test with exfoliated cells could be a powerful tool for detection of local genotoxic effects in humans, which is fundamental for hazard identification and risk estimation [13].

MN in peripheral blood lymphocytes has been extensively used to evaluate the presence and extend of chromosome damage in human populations exposed to genotoxic agents. As advantages, this MN test provides a reliable measure of chromosomal breakage and loss at lower cost and more easily than chromosomal aberrations. Moreover, the availability of cytokinesis-block technique eliminates potential background caused by effects on cell division kinetics [16].

The goal of this study is to contribute to the investigation of genotoxic effects in workers occupationally exposed to FA.

## Methods

### Subjects

This study was carried out in Portugal, in 80 workers occupationally exposed to FA vapors: 30 workers from FA and FA-based resins production factory and 50 from 10 pathology and anatomy laboratories. A control group of 85 non-exposed subjects was considered. All subjects were provided with the protocol and with the consent form, which they read and signed.

Health conditions, medical history, medication and lifestyle factors for all studied individuals, as well as information related to working practices (such as years of employment) were obtained through a standard questionnaire.

### Environmental Monitoring of FA exposure

Exposure assessment was performed by applying simultaneously two different methods.

In one of the methods, environmental samples were obtained by personal air sampling with low flow pumps during a typical working day. Sampling time was 6 to 8 hours. FA levels were measured by Gas Chromatography (GC) analysis and time-weighted average (TWA_8 h_) estimated according to the National Institute of Occupational Safety and Health method (NIOSH 2541) [17].

The other method was aimed to measure ceiling values of FA using Photo Ionization Detection (PID) equipment (11.7 eV lamps) with simultaneously video recording. Measures were performed in each task and instantaneous values for concentration were obtained on a per second basis. This method permits to establish a relation between worker activities and ceiling values and allows also determining the major emission sources [18].

### Micronucleus Test

Evaluation of genotoxic effects was performed by application of MN test in exfoliated cells from buccal mucosa and peripheral blood lymphocytes. Heparinized venous blood and exfoliated cells (buccal mucosa cells) were collected between 10 a.m. and 12 p.m., from each subject, and were processed for each test. All samples were coded and analyzed under blind conditions. The criterion of scoring the MN in lymphocytes is described in "The Human Micronucleus Project" and the buccal cells is described by *Tolbert et al*. [19,20].

### Buccal mucosa micronucleus test

Endobrush was used to collect cells from the buccal mucosa. Exfoliated cells were smeared onto the slides and fixed with Mercofix^®^. The standard protocol used was Feulgen staining technique without counterstain. Two thousand cells were scored from each individual by four independent observers. Only cells containing intact nuclei that were not clumped or overlapped were included in the analysis.

### Peripheral *Lymphocyte micronucleus test*

From each subject a blood specimen (10 mL) was collected using heparin as anticoagulant. The samples were kept refrigerated and processed within 6 hours of the blood collection. Lymphocytes were isolated using Ficoll-Paque gradient and placed in RPMI 1640 culture medium with L-glutamine and phenol red added with 10% inactivated fetal calf serum, 50 μg/mL streptomycin + 50 U/mL penicillin and 10 μg/mL phytohaemagglutinin. Duplicate cultures from each subject were incubated at 37°C in a humidified 5% CO_2 _incubator for 44 hours. Cytochalasin-b 6 μg/mL was added to cultures. After 28 hours of incubation, cells were spun onto microscope slides using a cytocentrifuge. Smears were air-dried and double stained with May-Grünwald-Giemsa and mounted with Entellan^®^.

A total of 1000 binucleated cells with well-preserved cytoplasm were examined for each donor. The frequencies of binucleated cells with MN were determined analyzing 1,000 binucleate lymphocytes from two slides for each subject.

### Statistical Analysis

Differences between groups (exposed workers and controls) were analyzed with *t*-Student and Proportion tests, in order to evaluate if basic characteristics of these 2 groups could be considered equivalent [20].

Association between quantitative variables was tested using correlation coefficient tests (Pearson or Spearman according with their probability distributions). Statistical analysis was performed with SPSS for Windows statistical package, version 17.0, and significance level was defined as 5%, for all inference studies.

## Results

### Characteristics of the studied population

The characterization of the population studied is summarized in Table 1. Controls and exposed workers did not differ significantly in age and in smoking habits. Only for gender distribution a significant difference was found between the two groups (*p *= 0.002), due to the larger number of women in the control group.

**Table 1:** Characterization of the studied population

	Control Group	Exposed Group	*P *value
Number of subjects	85	80	

Gender			
Male	31 (36.6%)	48 (60.0%)	0.002
Female	54 (63.5%)	32 (40.0%)	

Age (years)			
Range	20-55	19-56	
Mean	33.87	35.74	0.180
St. Deviation	8.262	9.470	0.024

Smoking status			
Non-smokers	59 (69.4%)	55 (68.8%)	0.927
Smokers	26 (30.6%)	25 (31.3%)	

Years of exposure			
Range	------	1 - 35	

None of the individuals presented relevant information about health conditions, medical history, medication and lifestyle factors that could influence the results of MN test.

### FA exposure levels

FA exposure levels obtained by two methods (NIOSH 2541 for average concentrations - TWA_8 h _and Photo Ionization Detection method for ceiling concentrations - C) are shown in Table 2.

**Table 2:** FA exposure in the two occupational settings

	Factory	Laboratories
Exposure duration (Years)		
Range	1 - 27	1 - 33
Mean	6.2	14.5
St. Deviation	6.74	9.12

Working hours/day (h)	7	7

Number of Samples (TWA measures)	2	29

FA exposure level (TWA_8 h_) (ppm)		
Range	0.20 - 0.22	0.05 - 0.51
Mean	0.21	0.28

FA ceiling concentration (ppm)		
Range	0.003 - 1.04	0.02 - 5.02
Mean	0.52	2.52

Time-weighted average concentrations (TWA_8 h_) have not exceeded the Occupational Safety and Health Administration (OSHA) reference value (0.75 ppm). On the other hand, ceiling concentrations were higher than American Conference of Industrial Hygienists (ACGIH) reference value (0.3 ppm) in both occupational settings.

Mean FA ceiling levels are higher in pathology and anatomy laboratories than in resins factory. In this setting, 83 tasks were studied and highest exposure level was observed during macroscopic examination of FA-preserved specimens. Moreover, 93% of studied tasks obtained ceiling levels higher than reference value (0.3 ppm). Pathologists were the professional group exposed to the highest ceiling concentration values (Table 3).

**Table 3:** FA Ceiling values (ppm) according to places of work, tasks and exposed workers

Places of work	Tasks	**Ceiling Values **(ppm)	Exposed Workers
Factory Resins production	Sample collect (Reactors)	1.09	Reactor operators

Factory Impregnation	Machine operation	1.04	Impregnation machine operators

Factory Quality Laboratory	Quality control	0.52	Quality Technicians

Pathology and anatomy laboratories	Macroscopic examination	5.02	Pathologist

Pathology and anatomy laboratories	Disposal of specimen and used solutions	0.95	Technicians and Assistants

Pathology and anatomy laboratories	Jar filling	2.51	Assistants

Pathology and anatomy laboratories	Specimen wash	2.28	Technicians

Pathology and anatomy laboratories	Biopsy	1.91	Technicians

Exposure has been studied in normal conditions of operation, namely with ventilation dispositives connected and workers not using protective masks.

### Micronucleus Test

The frequency of MN in occupationally exposed workers was significantly higher than in the control group, both in peripheral blood lymphocytes (*p *< 0.001) and in epithelial buccal cells (*p *< 0.001) (Table 4).

**Table 4:** Frequency of MN in the studied population

	Controls	Exposed		
		Factory	Pathology and anatomy laboratories	Total
MN PBL ^1^				
Mean ± Std. Dev	1.17 ± 1.95	1.76 ± 2.07	3.70 ± 3.86	2.97 ± 3.42

MN EBC^2^				
Mean ± Std. Dev	0.13 ± 0.48	1.27 ± 1.55	0.64 ± 1.74	0.88 ± 1.69

^1 ^peripheral blood lymphocytes (cytochalasin-B (binucleated) assay

^2 ^epithelial buccal cells

When analyzing each occupational setting separately, we found significant differences in MN frequencies in peripheral blood lymphocytes (*p *< 0.001) and in epithelial buccal cells (*p *< 0.005) between the laboratories and control groups. Concerning the factory group, significant differences in MN frequencies were only detected in epithelial buccal cells (*p *< 0.001).

Finally, we compared MN frequencies between the two exposed groups and found that MN frequency in peripheral blood lymphocytes was significantly higher in the laboratories group (*p *< 0.005), but respecting to epithelial buccal cells there was no significant difference between them (*p *= 0.108).

There was no significant difference in the frequency of MN, both in epithelial cells and peripheral lymphocyte tested for smoking habits (*p *= 0.31; *p *= 0.99) and for gender (*p *= 0.13; *p *= 0.47).

Age was found to have a weak positive correlation (r = 0.194; p < 0.05) with MN frequency in peripheral blood lymphocytes and a week negative correlation (r=-0.168; p < 0.05) with MN frequency in epithelial cells. A moderate positive correlation was found between duration of occupational exposure to FA (years of exposure) and frequency of MN in peripheral blood lymphocytes (r = 0.401; *p *< 0.05) and in epithelial cells (r = 0.209; *p *< 0.05) (Table 5).

**Table 5:** Correlation analysis between genotoxic endpoints and age and years of exposure (Spearman's test)

Genotoxic endpoints	Age	Years of Exposure
	r = 0.194	r = 0.401
MN PBL^1^	*p *= 0.013	*p *= 0.0

	r = - 0.168	r = 0.209
MN EBC^2^	*p *= 0.031	*p *= 0.008

^1 ^peripheral blood lymphocytes (cytochalasin-B (binucleated) assay)

^2 ^epithelial buccal cells

There was no significant difference (*p *> 0.05) in the frequency of MN in both peripheral blood lymphocytes and epithelial cells tested for smoking habits (*p *= 0.31; *p *= 0.99) and for gender (*p *= 0.13; *p *= 0.47).

## Discussion

As indicated by several studies [6,21,22] exposure assessment in present investigation identified that both groups of workers (factory and laboratory) were exposed to high peak FA concentrations.

The importance of this consideration lies in the fact that health effects (cancer) linked to FA exposure are more related with peaks of high concentrations than with long time exposure at low levels [2,23]. The choice of exposure metric should be based on the most biologically relevant exposure measure in order to diminish misclassification of exposure, thus leading to attenuated exposure-response relationships [24]. Moreover, exposures of short duration (peaks) are of special concern, because they produce an elevated dose rate at target tissues and organs, potentially altering metabolism, overloading protective and repair mechanisms and amplifying tissue responses [24,25].

Considering this, Pyatt *et al. *(2008) pointed out, as a limitation in most epidemiological studies, the lack of data about exposure to peak concentrations. Therefore, in those studies, health effects resulting from occupational exposure to FA are associated to exposure exclusively based on time-weighted average concentrations [23]. The only two studies concerning the association between exposure to FA and nasopharyngeal cancer that presented data on exposure to ceiling concentrations obtained higher relative risk values compared with the other studies [1,12,26].

Moreover, other groups also suggested ceiling concentrations as the most important exposure metric, when attempting to define the relative risk of myeloid leukaemia in workers exposed to FA [1,27-29].

The present results obtained in the laboratories, evidence a difference between the two exposure metrics (0.28 ppm for TWA_8 h _and 2.52 ppm for ceiling level). These results are in good agreement with previous ones reported by Shaham *et al. *[30]. A difference of the same order of magnitude was described in 14 pathology laboratories (0.4 ppm for TWA and 2.24 ppm for ceiling level).

Each one of these results would lead to different conclusions about exposure assessment and, consequently, to a different risk assessment and, though, claim our attention for the importance of exposure metric selection. For FA occupational exposure, ceiling concentrations might be a better strategy to evaluate exposure and to develop risk assessment once very high exposures over short periods are missed by TWA_8 h _method and, in fact, they are important to know the real risk for health [23,31]. Therefore, as in other investigations [33,34], it is possible to conclude that when measuring only TWA_8 h _poor information is obtained, and the method is of less utility to identify processes that should be targeted for controls.

Results indicate macroscopic examination of anatomical specimens FA-preserved as the task involving exposure to the highest values. This occurs because precision and good visibility is required and as a consequence pathologists must lean over the specimen with consequent increase of proximity to FA emission sources. Studies developed by Goyer *et al. *and Orsière *et al. *support that proximity to impregnated specimens promotes higher exposure to FA [6,22].

In factory, the task of collecting samples from resins reactor present the higher exposure, because the reactors are consider the units with larger emissions [6,32]. Furthermore, the sampling process is still manual in the factory studied.

It is important to notice that the information about exposure determinants, emission sources and exposed workers was only possible because video recording could be performed. This resource gives the opportunity to directly relate performance with exposure [18,35,36]. 

FA genotoxicity is confirmed in a variety of experimental systems ranging from bacteria to rodents in vivo [37]. Although the findings from *in vivo *animal studies may provide a basis for extrapolation to humans, cytogenetic assays in humans have been conflicting, sometimes with contradictory outcomes [38]. Nevertheless, our results showed a significant increase in MN frequency in epithelial cells and in lymphocytes of exposed individuals compared with controls.

Biological evidence of toxicity on distant-site such as peripheral lymphocytes and bone marrow is still controversial [2,39]. Some authors have argued that it is biologically implausible for FA to cause leukaemia as FA is unlikely to reach the bone marrow and cause toxicity. Due to its highly reactive nature and rapid metabolism, there is no evidence that it can damage stem and progenitor cells (the target cells for leukemogenesis). Also, there is no credible experimental animal model for FA-induced leukaemia [40,41]. However, Zhang *et al*. hypothesize that FA may act on bone marrow directly or, alternatively, may cause leukaemia by damaging the hematopoietic stem or early progenitor cells that are located in the circulating blood or nasal passages, which would then travel to bone marrow and become leukemic stem cells [1,29]. Nevertheless, our findings are consistent with other previous studies on epithelial cells and also on peripheral lymphocytes [42-44]. Suruda *et al. *reported that low-level exposure to FA was associated with cytogenetic changes in buccal epithelial cells and in blood lymphocytes in mortician students [14]. Our results in blood lymphocytes can be an indication that cytogenetic effects can be found in tissues distant from the area of initial contact (nasopharyngeal) and even reach the bone marrow and cause toxicity, supporting the thesis of Zhang and colleagues [1,29].

A significant positive correlation between MN frequency (both in peripheral blood lymphocytes and in epithelial buccal cells) and the duration of FA exposure (years of employment) was found (Table 4). This indicates that, together with peak contacts, exposure duration also has relevance for the development of health effects. Furthermore, in our study, long-term exposure to high levels of FA was noted particularly in pathology and anatomy laboratory workers (exposure duration mean of 14.5 years), fact that may at least contribute to explain the higher frequency of MN in peripheral blood lymphocytes in this group when compared to the factory group (Table 4). Regarding the influence of age, a positive correlation was found with MN frequency in peripheral blood lymphocytes (Table 5). MN frequencies tend to rise with age because of the progressive increase in spontaneous chromosome instability and the loss of efficiency in DNA repair mechanisms, which may result in accumulation of genetic lesions with increasing age [22,45]. On the other hand, for MN frequency in epithelial buccal cells, a negative correlation was found (Table 5). This can possibly be explained by the fact that cells of buccal mucosa have a steady and rapid turnover, and therefore accumulation of genotoxic effects becomes difficult [13].

No significant differences were obtained in MN frequencies between women and men (both in peripheral blood lymphocytes and epithelial buccal cells). However, in other studies an increase in MN frequencies in women was found. Current knowledge on the effect of gender on genetic damage determines a 1.5-fold greater MN frequency in females than in males [19,45], witch can be explained by preferential aneugenic events involving the X-chromossome. Surralés *et al. *reported an excessive overrepresentation of this chromosome in micronucleic lymphocytes cultured from women [46].

Tobacco smoke contains a high number of mutagenic and carcinogenic substances and is causally linked to an elevated incidence of several forms of cancers [47]. Hence, smoking is an important variable to consider in biomonitoring studies and, particularly in this study since FA is present in tobacco smoke [2]. The effect of tobacco smoking on MN frequency in human cells has been object of study. In most reports the results were unexpected, as in many instance smokers had lower frequencies of MN than non-smokers [22,48]. In the present study no significant differences were found in MN (peripheral blood lymphocytes and epithelial buccal cells) between smokers and non-smokers. These findings are similar to results obtained in the study of Bonassi *et al.*, [48]. These authors recommend that quantitative data about smoking habit should be collected because the sub-group of heavy smokers (≥30 cigarettes per day) can influence the results. For notice, the questionnaire results of this study revealed no heavy smokers in these workers groups.

## Conclusions

In conclusion, the population studied is exposed to high ceiling concentrations (peaks) of FA with a long-term exposure. These two aspects, cumulatively, can be the cause for the increase in MN frequencies in lymphocytes and in epithelial buccal cells.

Results obtained suggest that preventive and protective measures must be applied in order to reduce occupational exposure to this chemical agent in these two occupational settings and, subsequently, to prevent adverse effects on workers health.

## Competing interests

The authors declare that they have no competing interests.

## Authors' contributions

SV conceived the idea and designed the study, and developed also formaldehyde exposure assessment. CL, PM, JMV and MB performed the MN tests. MG developed chemical analysis. CN performed the statistical data analyses. JP contributed to the study design and coordination. All authors have read and approved the final manuscript.
